# To Professional Correspondents

**Published:** 1843-01

**Authors:** 


### TO PROFESSIONAL CORRESPONDENTS.

To prevent disappointment, we beg leave to say to such of our readers as may have occasion to address us on professional subjects, that as soon as our winter labours in the Institute can be brought to a close, we shall descend to the Gulf of Mexico, to spend several months in a medical examination of the maritime parts of Louisiana, Mississippi, Alabama and Florida. Should any of them, however, have a special reason for addressing us, the letter, if sent to Cincinnati, will be forwarded in such direction as to reach us. All communications designed for the Journal, must be directed to its Editors, and the superscription need not include the name of either.

Offering to our readers the compliments of the season, and hoping that their ledgers, on the 1st of January, may show fewer outstanding balances than generally disfigure and oppress a doctor's books, we bid them FAREWELL! D.

